# The molecular genetics of RASopathies: An update on novel disease genes and new disorders

**DOI:** 10.1002/ajmg.c.32012

**Published:** 2022-11-16

**Authors:** Marco Tartaglia, Yoko Aoki, Bruce D. Gelb

**Affiliations:** ^1^ Genetics and Rare Diseases Research Division Ospedale Pediatrico Bambino Gesù, IRCCS Rome Italy; ^2^ Department of Medical Genetics Tohoku University School of Medicine Sendai Japan; ^3^ Mindich Child Health and Development Institute Icahn School of Medicine at Mount Sinai New York New York USA; ^4^ Department of Pediatrics and Genetics Icahn School of Medicine at Mount Sinai New York New York USA; ^5^ Department of Genomic Sciences Icahn School of Medicine at Mount Sinai New York New York USA

**Keywords:** LZTR1, MAPK1, MRAS, RAS signaling, RRAS2, SPRED2

## Abstract

Enhanced signaling through RAS and the mitogen‐associated protein kinase (MAPK) cascade underlies the RASopathies, a family of clinically related disorders affecting development and growth. In RASopathies, increased RAS‐MAPK signaling can result from the upregulated activity of various RAS GTPases, enhanced function of proteins positively controlling RAS function or favoring the efficient transmission of RAS signaling to downstream transducers, functional upregulation of RAS effectors belonging to the MAPK cascade, or inefficient signaling switch‐off operated by feedback mechanisms acting at different levels. The massive effort in RASopathy gene discovery performed in the last 20 years has identified more than 20 genes implicated in these disorders. It has also facilitated the characterization of several molecular activating mechanisms that had remained unappreciated due to their minor impact in oncogenesis. Here, we provide an overview on the discoveries collected during the last 5 years that have delivered unexpected insights (e.g., Noonan syndrome as a recessive disease) and allowed to profile new RASopathies, novel disease genes and new molecular circuits contributing to the control of RAS‐MAPK signaling.

## 1 THE GENETICS OF NOONAN SYNDROME AND CLINICALLY RELATED DISORDERS: AN HISTORICAL PERSPECTIVE

In 2001, *PTPN11* (MIM: 176876), encoding SHP2, a non‐receptor protein tyrosine phosphatase having a relevant role in intracellular signaling and various developmental processes (Tajan, de Rocca Serra, Valet, Edouard, & Yart, 2015; Tartaglia & Gelb, 2005), was identified as the major Noonan syndrome (NS, MIM: PS163950) disease gene using a positional candidacy approach (Tartaglia et al., 2001). In the ensuing years, studies performed by the same team and several others provided evidence that pathogenic variants in this gene account for approximately 50% of NS, defined the mutational spectrum characterizing the disorder, demonstrated their activating role on SHP2's functional dysregulation, and established clinically relevant genotype–phenotype associations (Fragale, Tartaglia, Wu, & Gelb, 2004; Tartaglia et al., 2002; Zenker et al., 2004). Following this key discovery, a distinct set of *PTPN11* mutations was identified in individuals with NS with multiple lentigines (NSML, MIM: PS151100), also known as LEOPARD syndrome (Digilio et al., 2002; Legius et al., 2002), a developmental disorder known to be closely related to NS, providing first evidence of allelic heterogeneity for *PTPN11*.

At that time, clinical reports had highlighted a specific association between NS and myeloproliferative disorders, including juvenile myelomonocytic leukemia (JMML; MIM: 607785; Bader‐Meunier et al., 1997; Choong et al., 1999). In 2003, based on that association, missense mutations in *PTPN11* were discovered as somatic events in JMML, as well as in childhood myelodysplastic syndromes (MDS) and acute myeloid leukemia (AML; Tartaglia et al., 2003). This work also identified a subset of germline variants in this gene specifically predisposing to JMML in NS. Subsequent studies further expanded the contribution of somatic *PTPN11* mutations in malignancies (Loh et al., 2004; Tartaglia et al., 2004, 2005), demonstrating their specific relevance in the context of childhood neoplasias (Bentires‐Alj et al., 2004; Hugues et al., 2005; Johan et al., 2004; Loh et al., 2005; Watkins, Fidler, Boultwood, & Wainscoat, 2004). Notably, those studies elucidated the different spectrum of NS‐causing and leukemia‐associated mutations, suggesting a differential quantitative perturbing effect of the two classes of mutations in upregulating SHP2's function and RAS‐MAPK signaling. These findings also provided evidence supporting the notion that upregulation of RAS‐MAPK signaling, a signal transduction cascade acting as a well‐known driver event in oncogenesis when strongly upregulated, may be compatible with embryogenesis and development and underlie a developmental disorder when mildly upregulated (Tartaglia et al., 2003). This working hypothesis was confirmed by studies that functionally characterized these classes of mutations, demonstrating their direct impact on RAS‐MAPK signaling (R. J. Chan et al., 2005; Fragale et al., 2004; Keilhack, David, McGregor, Cantley, & Neel, 2005; Schubbert et al., 2005; Tartaglia et al., 2006). This unpredicted scenario was further confirmed by the identification of activating pathogenic variants of *HRAS*, a proto‐oncogene frequently mutated in cancer, as the molecular cause of Costello syndrome (CS; MIM 218040), a disorder related to NS characterized by an overall more severe clinical presentation and predisposition to certain malignancies (Aoki et al., 2005).

**FIGURE 1:**
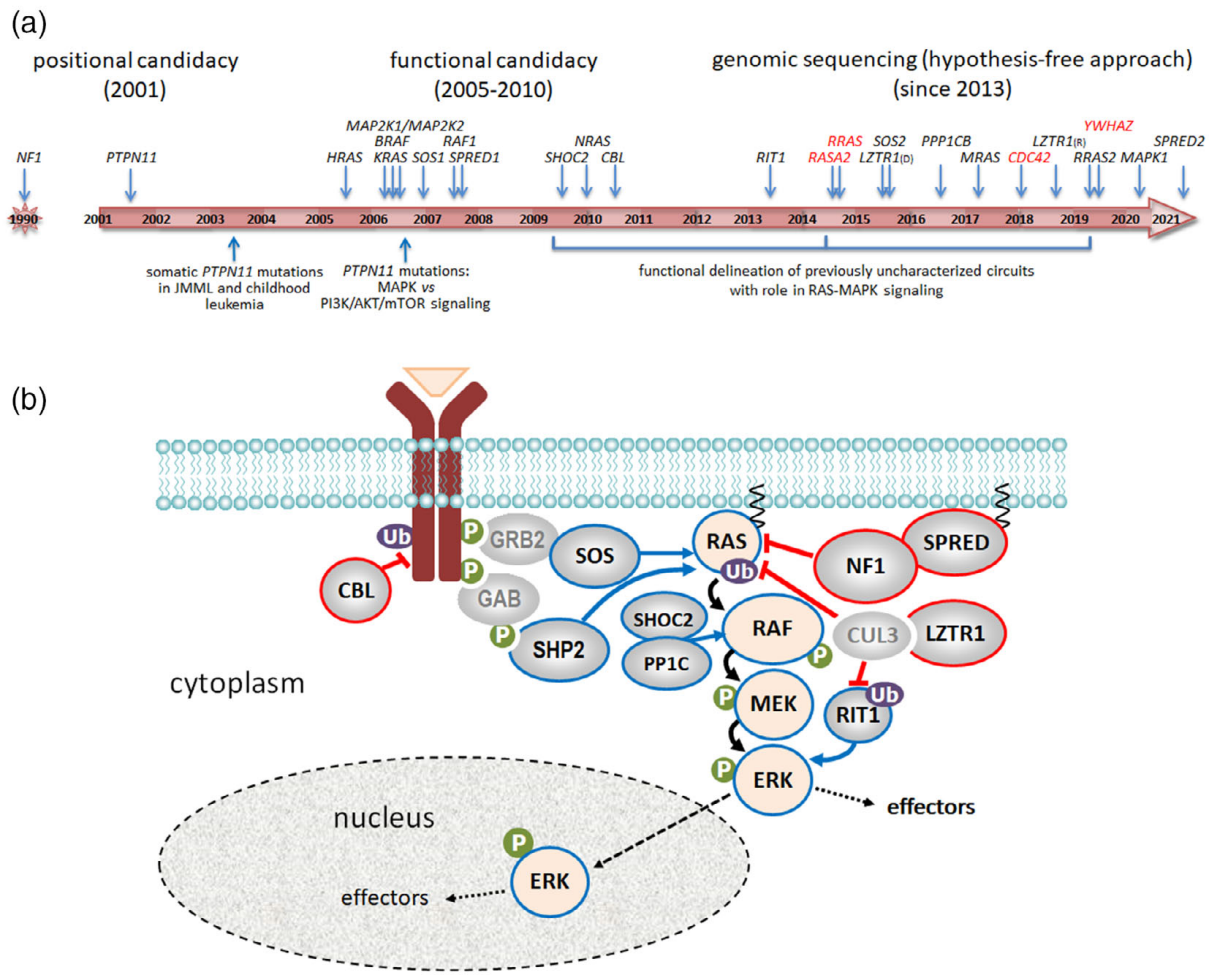
RASopathy genes and the role of their encoded proteins in the RAS‐MAPK signaling cascades. (a) Cartoon listing the genes implicated in RASopathies and their year of discovery. Genes requiring further clinical delineation and/or functional validation in the context of the RASopathies are shown in red. Milestones linked to these discoveries are also reported. D and R indicate dominant and recessive, respectively. (b) The RAS‐MAPK signaling cascade. The cartoon shows the signal flow through the pathway (black arrows), together with the proteins positively (blue) and negatively (red) controlling the cascade. Signaling upregulation occurring in RASopathies results from enhanced activity of RAS proteins (i.e., HRAS, KRAS, NRAS, MRAS, RRAS, RRAS2, and RIT1), upstream positive signal transducers and regulators (i.e., SHP2, SOS1, and SOS2), proteins favoring transmission of RAS signaling to downstream transducers (i.e., MRAS, SHOC2, and PPP1CB), and tiers of the MAPK cascade (i.e., BRAF, RAF1, MAP2K1, MAP2K2, and MAPK1). Signaling upregulation also results from inefficient signaling switch‐off operated by multiple feedback mechanisms (i.e., defective/impaired function of CBL, neurofibromin, LZTR1, SPRED1, and SPRED2). P and Ub indicate phosphorylation and ubiquitination, respectively.

Based on those pioneering discoveries, a massive screening effort using functional candidacy (i.e., mutation scan of genes with role in RAS signaling and downstream pathways, including the MAPK cascade) was performed in the subsequent 5 years, resulting in the identification of several disease genes implicated in NS (*KRAS* [MIM: 190070], *SOS1* [MIM: 182530], *RAF1* [MIM: 164760], *BRAF* [MIM: 164757], *MAP2K1* [MIM: 176872], and *NRAS* [MIM: 164790]) and cardiofaciocutaneous syndrome (CFCS; MIM PS 115150; *KRAS*, *BRAF*, *MAP2K1*, and *MAP2K2* [MIM: 601263]; Figure 1, Table 1; Tartaglia, Gelb, & Zenker, 2011; Aoki & Matsubara, 2013; Rauen, 2013). This activity resulted in the clinical and molecular definition of new related disorders, including Legius syndrome [MIM: 611431], Mazzanti syndrome, also known as Noonan syndrome‐like disorder with loose anagen hair (MIM: PS607721) and CBL syndrome (MIM: 613563), respectively caused by mutations in the *SPRED1* (MIM: 609291), *SHOC2* (MIM: 602775) and *CBL* (MIM: 165360) genes (Brems et al., 2007; Cordeddu et al., 2009; Martinelli et al., 2010; Niemeyer et al., 2010; Pérez et al., 2010). Of note, before the introduction of massive parallel sequencing, functional candidacy represented the only available gene hunting strategy since no sufficiently informative *PTPN11* mutation‐negative family had been identified to support a genetic characterization driven through linkage analysis and positional candidacy. This new knowledge enabled more precise characterization of the clinical variability and genetic heterogeneity of NS and CFCS but also an appreciation of the occurrence of a shared molecular basis underlying the clinical overlap of these developmental disorders, which are currently collectively known as “RASopathies". Even though an autosomal recessive form of NS had been suggested (van der Burgt & Brunner, 2000), these findings documented an apparently invariant autosomal dominant inheritance pattern for these diseases, which has only recently been reconsidered (Johnston et al., 2018; Motta et al., 2021).

**TABLE 1:** List of genes implicated in Noonan syndrome and clinically related disorders.

Gene (OMIM ID)	Protein function in RAS‐MAPK signaling	RASopathy (OMIM ID)	Notes (OMIM ID)	Reference
*CBL* (165360)	Negative regulator (Ub)	CBL‐related RASopathy (613563)	JMML	1–3
*PTPN11* (176876)	PTP (multiple substrates)	NS (163950) NSML (151100)	JMML; pediatric cancers; PVS, via MAPK upregulation (NS) HCM, via PI3K‐AKT upregulation (NSML)	4–6 7–9
*SOS1* (182530)	GEF	NS (610733)	Genotype–phenotype correlations	10,11
*SOS2* (601247)	GEF	NS (616559)	Genotype–phenotype correlations	12,13
*HRAS* (190020)	RAS GTPase	CS (218040)	Cancer predisposition, mosaic RASopathies (162900, 163200, 137550)	14
*KRAS* (190070)	RAS GTPase	Wide RASopathy spectrum (615278, 609942)	Postzygotic events involving oncogenic variants in mosaic RASopathies (108010, 600268, 163200)	15–17
*NRAS* (164790)	RAS GTPase	NS (613224)	Postzygotic events involving oncogenic variants in mosaic RASopathies (162900, 137550, 163200)	18
*NF1* (613113)	GAP	NF1 (162200, 601321, 193520)	Cancer predisposition	19
*LZTR1* (600574)	Negative control (Ub)	NS (616564, 605275) Schwannomatosis (615670)	Dominant and recessive forms	2,20–22 23
*SPRED1* (609291)	Negative control (RAS)	LS (611431)		24
*SPRED2* (609292)	Negative control (RAS)	NS (619745)	Recessive form	25
*RIT1* (609591)	RAS GTPase	NS (615355)	HCM	26
*RRAS2* (600098)	RAS GTPase	NS (618624)		27,28
*SHOC2* (602775)	Positive regulator (RAF1)	NSLAH (607721)	Narrow mutational spectrum, p.Ser2Gly, >95% of cases	29
*MRAS* (608435)	RAS GTPase	NS (618499)	HCM	30,31
*PPP1CB* (600590)	SPP (RAF1/BRAF)	NSLAH (617506)		32
*RAF1* (164760)	Kinase (MAP2K1/2)	NS (611553)	HCM	33,34
*BRAF* (164757)	Kinase (MAP2K1/2)	CFCS (115150)	Wide RASopathy spectrum (613707, 613706)	16,35,36
*MAP2K1* (176872)	Kinase (MAPK1/2)	CFCS (615279)	NS, rarely; melorheostosis (155950)	35,37
*MAP2K2* (601263)	Kinase (MAPK1/2)	CFCS (615280)		35,37
*MAPK1* (176948)	Kinase (multiple substrates)	MAPK1‐related RASopathy (619087)		38

*Note*: References: 1, Martinelli et al., 2010; 2, Niemeyer et al., 2010; 3, Pérez et al., 2010; 4, Tartaglia et al., 2001; 5, Tartaglia et al., 2002; 6, Tartaglia et al., 2003; 7, Digilio et al., 2002; 8, Legius et al., 2002; 9, Hanna et al., 2006; 10, Tartaglia et al., 2006; 11, Roberts et al., 2007; 12, Yamamoto et al., 2015; 13, Cordeddu et al., 2015; 14, Aoki et al., 2005; 15, Schubbert et al., 2006; 16, Niihori et al., 2006; 17, Carta et al., 2006; 18, Cirstea et al., 2010; 19, Wallace et al., 1990; 20, Johnston et al., 2018; 21, Motta et al., 2019; 22, Pagnamenta et al., 2019; 23, Piotrowski et al., 2014; 24, Brems et al., 2007; 25, Motta et al., 2021; 26, Aoki et al., 2013; 27, Capri et al., 2019; 28, Niihori et al., 2019; 29, Cordeddu et al., 2009; 30, Higgins et al., 2017; 31, Motta et al., 2020; 32, Gripp et al., 2016; 33, Pandit et al., 2007; 34, Razzaque et al., 2007; 35, Rodriguez‐Viciana et al., 2006; 36, Sarkozy et al., 2009; 37, Nava et al., 2007; 38, Motta et al., 2020.

Abbreviations: CFCS, cardiofaciocutaneous syndrome; CS, Costello syndrome; GAP, GTPase activating protein; GEF, guanine nucleotide exchange factor; HCM, high prevalence of hypertrophic cardiomyopathy; JMML, predisposition for juvenile myelomonocytic leukemia; LS, Legius syndrome; NF1, neurofibromatosis type I; NS, Noonan syndrome; NSLAH, Mazzanti syndrome (aka Noonan syndrome‐like disorder with loose anagen hair); NSML, LEOPARD syndrome (aka Noonan syndrome with multiple lentigines); SPP, serine/threonine‐specific protein phosphatase; PTP, protein tyrosine phosphatase; PVS, high prevalence of pulmonary valve stenosis; Ub, ubiquitination of signal transducers.

During the last 10 years, the use of hypothesis‐free disease gene discovery approaches based on genome‐wide sequencing has facilitated the identification of additional RASopathy genes (Figure 1, Table 1). Some of them had already been recognized as relevant signal transducers or modulators of RAS proteins and the MAPK cascade (e.g., *SOS2* [MIM: 601247]); on the other hand, the functional link to this signaling network was not obvious for others (e.g., *LZTR1* [MIM: 600574]). In this review, we briefly outline the molecular mechanisms affecting RAS‐MAPK signaling in RASopathies and provide an overview on the novel disease genes and new RASopathies emerged in the last few years.

## 2 MOLECULAR MECHANISMS PERTURBING RAS‐MAPK SIGNALING IN RASOPATHIES: COMMON THEMES AND NEW PLAYERS

RAS proteins are small guanosine triphosphate (GTP)/guanosine diphosphate (GDP)‐binding GTPases functioning as molecular switches that control diverse cellular processes (e.g., cell fate determination, proliferation, survival, differentiation, migration, and senescence) by modulating a multifaceted signaling network (Cox, Fesik, Kimmelman, Luo, & Der, 2014; Pylayeva‐Gupta, Grabocka, & Bar‐Sagi, 2011; Simanshu, Nissley, & McCormick, 2017). RAS proteins are activated in response to the binding of extracellular ligands (e.g., growth factors) to cognate cell surface receptors, which promotes the recruitment of guanine nucleotide‐exchange factors (GEFs) to the plasma membrane and their binding to RAS proteins, favoring the release of GDP and binding to the more prevalent GTP in the latter. Activated GTP‐bound RAS interact with different effector proteins promoting signal flow (Simanshu et al., 2017). The mitogen‐activated protein kinase (MAPK) cascade (Figure 1), which is a major signaling pathway downstream of RAS (Klomp, Klomp, & Der, 2021; Yoon & Seger, 2006), is activated by the recruitment of the RAF serine/threonine kinases (RAF1, BRAF, and ARAF) to the cytoplasmic surface of cellular membranes, favoring their catalytic activation. In turn, the first tiers of this cascade phosphorylate and activate their substrates, the dual specificity MAPK/ERK kinases (MEK1 and MEK2). Upon activation, MEK proteins phosphorylate regulatory residues of the extracellular signal‐regulated kinases (ERK1 and ERK2), which are serine/threonine protein kinases that modulate the activity of a large number of cytoplasmic and nuclear substrates. Multiple signaling platforms and feedback mechanisms control the specificity and extent of signal flow through this pathway at different levels (Klomp et al., 2021; Kolch, 2005; Lake, Corrêa, & Müller, 2016), including the functional inactivation of RAS by GTPase activating proteins (GAPs), which stimulate their intrinsically low GTPase activity.

Pathogenic variants in more than 20 genes encoding signal transducers or regulators with role in the RAS‐MAPK signaling network have been established to underlie RASopathies (Capri et al., 2019; Grant et al., 2018; Motta et al., 2021; Motta, Pannone, et al., 2020; Niihori et al., 2019). In these disorders, increased signaling through RAS and the MAPK cascade can result from upregulated activity of RAS proteins (i.e., HRAS, KRAS, NRAS, MRAS, RRAS, RRAS2, and RIT1), enhanced function of upstream signal transducers and regulators positively controlling RAS function (i.e., SHP2, SOS1, and SOS2) or downstream proteins favoring the efficient transmission of RAS signaling to downstream transducers (i.e., MRAS, SHOC2, PPP1CB), RAS effectors belonging to the MAPK cascade (i.e., BRAF, RAF1, MAP2K1, MAP2K2, MAPK1), as well as from the inefficient signaling switch‐off by feedback mechanisms acting at different levels converging to down‐modulate RAS function (i.e., CBL, LZTR1, neurofibromin, SPRED1, and SPRED2; Figure 1).

A remarkable finding that has emerged in dissecting the molecular causes of RASopathies is the occurrence of conserved themes in the mechanism of disease. This applies to mutations affecting genes encoding the various members of the RAS superfamily of GTPases that have been implicated in these disorders (Aoki et al., 2005; Capri et al., 2019; Cirstea et al., 2010; Flex et al., 2014; Higgins et al., 2017; Motta, Sagi‐Dain, et al., 2020; Niihori et al., 2019; Schubbert et al., 2006). These missense mutations generally affect a small number of highly conserved amino acid residues that lead to hyperactivation of these proteins by decreasing/impairing their GTPase activity in response to GTPase activating proteins (GAPs) or increasing guanine nucleotide exchange factor (GEF)‐independent GDP release. Remarkably, as anticipated for NS‐causing and leukemia‐associated *PTPN11* mutations, the germline mutations affecting these genes may involve the same residues that represent hot spots in cancer, but the former are generally less activating than the latter.

The massive effort in RASopathy gene discovery has facilitated the identification of a number of new molecular mechanisms dysregulating RAS‐MAPK signaling that had remained unappreciated due to their negligible impact in oncogenesis. It is also worth noting that most RASopathy‐causing mutations show functional convergence operating at the level of RAS and RAF proteins. Indeed, the defective function of an increasing number of proteins (i.e., neurofibromin, LZTR1, SPRED1 and SPRED2) provides evidence of multiple circuits implicated in the negative control of RAS function as a recurrent theme of RAS‐MAPK signaling upregulation in RASopathies (Bergoug et al., 2020; Brems et al., 2007; Motta et al., 2021; Piotrowski et al., 2014). A second shared leitmotif includes different mechanisms favoring a more stable GTP‐bound RAS‐RAF1 interaction, which is a key step required for efficient MAPK activation. Indeed, the major mutation cluster identified in *RAF1* (>80% of NS‐causing mutations; Pandit et al., 2007), the almost invariant missense change in *SHOC2* (p.Ser2Gly; Cordeddu et al., 2009), and the narrow spectrum of pathogenic variants in *PPP1CB* (Gripp et al., 2016) and *MRAS* (see below), functionally converge to favor the dephosphorylated status of RAF1 at Ser^259^, which is a crucial step in RAF1 catalytic activation (Young et al., 2018).

## 3 THE LAST 5 YEARS: NOVEL RASOPATHY GENES, NEW RASOPATHIES AND MORE

The clinical and molecular aspects of RASopathies have been outlined by a number of dedicated reviews (Aoki, Niihori, Inoue, & Matsubara, 2016; Rauen, 2013; Tajan, Paccoud, Branka, Edouard, & Yart, 2018; Tartaglia & Gelb, 2010; Tidyman & Rauen, 2016). Here, we provide an overview on the discoveries collected during the last 5 years, which have delivered unexpected insights (e.g., NS as a recessive disease) and allowed to profile new RASopathies, novel disease genes, and new molecular circuits contributing to the control of RAS‐MAPK signaling. Only genes with robust and definitely established clinical link to RASopathies are here discussed.

### 3.1 Enhanced MAPK1 function causes a new neurodevelopmental disorder within the RASopathy clinical spectrum

Two years ago, nearly 20 years from the discovery of *PTPN11* as the first gene implicated in NS, the mitogen‐activated protein kinase 1 gene (*MAPK1*, MIM: 176948), also known as extracellular signal‐regulated protein kinase 2 (*ERK2*), encoding the terminal tier of the MAPK cascade, joined the group of the signal transducers mutated in the RASopathies.

As an effector of the MAPK pathway, MAPK1/ERK2 modulates the activity of hundreds of cytoplasmic and nuclear proteins (Ünal, Uhlitz, & Blüthgen, 2017). Inactive ERK is located in the cytoplasm; upon activation, it translocates to the nucleus, regulating gene expression. The catalytic activity of this ubiquitously expressed protein serine/threonine kinase requires MEK‐mediated phosphorylation of two regulatory phosphorylatable residues, Thr^185^ and Tyr^187^, which are located within an “activation” segment, favoring a conformation of the catalytic domain stabilizing the active site (Roskoski Jr., 2012; Rubinfeld & Seger, 2005). Dephosphorylation of Thr^185^ and Tyr^187^ is catalyzed by MAPK phosphatases (MKPs) and is a required step for the inactivation of the kinase. The reversible phosphorylated state of these two residues controls the magnitude and duration of activation as well as the nuclear localization of the kinase, which, in turn, determines the nature of the cellular response to stimuli. Specificity in substrate binding is mainly attained by interactions mediated by two “docking sites,” named D‐recruitment site (DRS) and F‐recruitment site (FRS; Peti & Page, 2013; Reményi, Good, & Lim, 2006). The DRS is located behind the ATP‐binding pocket and is engaged by partners that contain a “D‐site” consensus sequence, while the FRS is located below the activation loop and preferentially binds partners that contain a consensus “F‐site” sequence (Ghose, 2019).

De novo *MAPK1* variants promoting hyperactivation of the kinase cause a neurodevelopmental disorder within the RASopathy phenotypic spectrum (Motta, Pannone, et al., 2020; Motta, Sagi‐Dain, et al., 2020). While variable in terms of severity, the clinical phenotype of the seven subjects reported so far includes developmental delays and intellectual disabilities (DD/ID), commonly associated with behavioral problems (Table 2). Postnatally reduced growth occurs in approximately half of affected individuals. Craniofacial anomalies, including hypertelorism, ptosis, low‐set/posteriorly rotated ears with a distinctive morphology, wide nasal bridge, are also common. In some subjects, the facies and co‐occurrence of a short/webbed neck, low posterior hairline, skin features (e.g., multiple lentigines, *cafè au lait* spots [CALS]) and reduced growth are suggestive of NS. Congenital heart defects (e.g., atrial septal defects and mitral valve insufficiency) were reported in a relatively high proportion of patients, although hypertrophic cardiomyopathy (HCM) has not been reported. Minor skeletal defects were also a common finding. None of the subjects has been diagnosed with cancer.

**TABLE 2:** Clinical features associated with pathogenic variants in *RRAS2*, *MRAS*, *LZTR1*, *SPRED2*, and *MAPK1*

	Noonan syndrome					
	*RRAS2*	*MRAS*	*LZTR1* (D)	*LZTR1* (R)	*SPRED2*	*MAPK1*
No. affected subjects (F, M)	13 (6, 7)	6 (3, 3)	32 (18, 14)	31 (16, 15)	4 (2, 2)	7 (1, 6)
Growth						
Macrocephaly (>2SD)	4/12 (33%)	0/6	0/32	0/31	0/4	1/7 (14%)
Microcephaly (<2SD)	1/12 (8%)	0/6	3/32 (10%)	2/31 (6%)	0/4	3/7 (43%)
Short stature (<2SD)	4/12 (33%)	3/6 (50%)	23/32 (72%)	15/31 (48%)	2/4 (50%)	4/7 (57%)
Facies						
Typical/suggestive facies			32/32 (100%)	31/31 (100%)		
Hypertelorism	11/12 (92%)	5/6 (83%)	19/25 (76%)	10/31 (32%)	3/4 (75%)	5/7 (71%)
Ptosis	11/12 (92%)	6/6 (100%)	17/23 (74%)	11/31 (35%)	3/4 (75%)	5/7 (71%)
Down‐slanting palpebral fissures	11/12 (92%)	6/6 (100%)	16/23 (70%)	14/31 (45%)	3/4 (75%)	3/7 (43%)
Low‐set/posteriorly rotated ears	12/12 (100%)	4/6 (67%)	10/12 (83%)	17/31 (55%)	3/4 (75%)	6/7 (86%)
Wide nasal bridge	12/12 (100%)	5/6 (83%)	11/17 (65%)	12/31 (39%)	4/4 (100%)	3/7 (43%)
Low posterior hairline	3/12 (25%)	1/6 (17%)	6/32 (19%)	12/31 (39%)	4/4 (100%)	4/7 (57%)
Short/webbed neck	3/12 (25%)	3/6 (50%)	15/32 (47%)	21/31 (68%)	4/4 (100%)	4/7 (57%)
Development						
DD	5/11 (45%)	4/6 (67%)	5/32 (16%)	13/31 (42%)	3/4 (75%)	7/7 (100%)
Language delay	4/11 (36%)				3/4 (75%)	6/7 (86%)
Learning disorder					4/4	7/7 (100%)
ID		2/6 (33%)	9/32 (28%)	11/31 (35%)	(100%)	6/7 (86%)
Behavioral problems					3/4 (75%)	7/7 (100%)
Neurological features						
Hypotonia					3/4 (75%)	4/7 (57%)
Epilepsy			2/32 (6%)		0/4	2/7 (29%)
Cardiac involvement						
PS	1/13 (8%)	1/6 (17%)	8/32 (25%)	3/31 (10%)	2/4 (50%)	0/7
ASD	4/13 (31%)	2/6 (33%)	5/32 (16%)	2/31 (6%)	1/4 (25%)	2/7 (29%)
MVP			3/32 (9%)		0/4	3/7 (43%)
HCM		6/6 (100%)	6/32 (19%)	21/31 (68%)	2/4 (50%)	0/7
Others (not specified)	3/13 (23%)	1/6 (17%)	5/32 (16%)	19/31 (61%)		
Skeletal anomalies						
Broad thorax	1/13 (8%)		4/32 (13%)	15/31 (48%)	2/4 (50%)	2/7 (29%)
Pectus deformities	3/13 (23%)	2/6 (33%)	18/32 (56%)	12/32 (38%)	4/4 (100%)	0/7
Pes planus				2/31 (6%)	1/4 (25%)	3/7 (43%)
Skin features						
CALS			4/32 (13%)		0/4	2/7 (29%)
Multiple lentigines					0/4	1/7 (14%)
Freckling					0/4	1/7 (14%)
Dry skin/eczema					1/4 (25%)	2/7 (29%)
Hypertrichosis			2/32 (6%)		0/4	2/7 (29%)
Cryptorchidism	3/7 (43%)		5/14 (36%)	4/15 (27%)	1/2 (50%)	2/6 (33%)
Bleeding/easy bruising	1/13 (8%)		6/32 (19%)	1/31 (3%)	2/4 (50%)	1/7 (14%)
Others	JMML (1)			Malignancies (2)^a^		

*Note*: References: Capri et al., 2019, Niihori et al., 2019, Weinstock & Sadler, 2022 (*RRAS2*); Higgins et al., 2017, Suzuki et al., 2019, Motta, Sagi‐Dain, et al., 2020, Pires et al., 2021 (*MRAS*); Yamamoto et al., 2015, Pagnamenta et al., 2019, Güemes et al., 2019, Umeki et al., 2019, Jacquinet et al., 2020, Zhao et al., 2021, Farncombe, Thain, Barnett‐Tapia, Sadeghian, & Kim, 2022 (*LZTR1*, dominant NS); Johnston et al., 2018, H. Chen et al., 2019, Perin et al., 2019, Pagnamenta et al., 2019, Umeki et al., 2019, Nakagama et al., 2020 (*LZTR1*, recessive NS); Motta et al., 2021 (*SPRED2*); Motta, Pannone, et al., 2020 (*MAPK1*).

Abbreviations: ASD, atrial septal defects; CALS, café‐au‐lait spots; D, dominant; DD, developmental delay; F, females; HCM, hypertrophic cardiomyopathy; ID, intellectual disability; M, males; MVP, mitral valve prolapse; PVS, pulmonic stenosis; R, recessive.

^a^ Leukemia, unspecified (*N* = 1); optic glioma (*N* = 1).

Pathogenic *MAPK1* variants have a non‐random distribution and promote an increased phosphorylation of the kinase, which in turn enhances the translocation of the protein to the nucleus and boosts MAPK signaling (Motta, Pannone, et al., 2020; Motta, Sagi‐Dain, et al., 2020). Of note, the collected data indicate that these variants can be classified within two mechanistic groups affecting either binding of the kinase to regulators and effectors (amino acid substitutions at residues His^80^, Asp^318^, Glu^322^, and Pro^323^) or perturbing the regulatory mechanism controlling the activation of the kinase (changes at residues Ile^74^ and Ala^174^). Functional studies showed that the defective negative regulation exerted by MKP3 (also known as DUSP6), a dual‐specificity protein phosphatase negatively controlling ERK function, represents a generalizable mechanism for the gain‐of‐function (GoF) behavior of these pathogenic variants. Of note, previous work demonstrated that amino acid substitutions in or close to the DRS can lead to either GoF or loss of function (LoF) by impairing proper MAPK1 binding to regulators and substrates, respectively (Brenan et al., 2016). The available structural and experimental data support a model in which disease‐causing variants may operate with counteracting effects on MAPK1 function by differentially impacting the ability of the mutated protein to interact with MAP2K1/2, MKP3 and substrates (Motta, Pannone, et al., 2020; Motta, Sagi‐Dain, et al., 2020).

As a recurrent theme in RASopathies, signal dysregulation driven by pathogenic MAPK1 variants is stimulus reliant. In this case, the hyperactive function of MAPK1 retains dependence on MEK activity. Remarkably, while enhanced MAPK1 activation associated with oncogenic mutations in upstream signal transducers (e.g., KRAS, HRAS, NRAS, and BRAF) is a common finding in cancer, *MAPK1* mutations do not represent a major somatic event contributing to oncogenesis. This finding and the observation that mutations likely have counteracting effects possibly explains the minor role of these variants as driver events contributing to oncogenesis. This is presumably due to the particular behavior of these mutations, which weaken MKP3 binding and prejudice MAPK1 dephosphorylation of the regulatory residues, Thr^185^ and Tyr^187^, but also may variably impact binding of the kinase to a large number of interacting proteins, including substrates (Brenan et al., 2016; Canagarajah, Khokhlatchev, Cobb, & Goldsmith, 1997; Roskoski Jr., 2015).

Overall, de novo *MAPK1* mutations define a new RASopathy. Although two individuals of the original cohort had features suggestive of NS, systematic screening of the entire *MAPK1* coding sequence in large cohorts of subjects with clinical diagnosis of RASopathy did not identify other pathogenic variants. This negative result suggests that *MAPK1* mutations are a rare event in subjects with NS‐related features and could be more commonly identified in patients with unclassified syndromic DD/ID.

### 3.2 Two classes of LZTR1 mutations underlie the dominant and recessive forms of Noonan syndrome

In 2014, *LZTR1* (MIM: 600574) was identified as a gene predisposing to schwannomatosis (MIM: 162091; Piotrowski et al., 2014), an adult‐onset tumor predisposition disease clinically and genetically distinct from neurofibromatosis type 1 (MIM: 162200) and type 2 (MIM: 101000). A wide spectrum of pathogenic variants was identified, including missense, nonsense, frameshift and splice site changes, spotted throughout the entire coding sequence. One year later, heterozygous germline variant in the same gene were discovered to cause NS (Yamamoto et al., 2015). Similar missense variants had previously been reported in two additional subjects with NS but were not considered as clinically relevant (P. C. Chen et al., 2014). Intriguingly, all NS‐associated variants were reported as missense changes clustering at the N‐terminus of the protein, suggesting a specific functional impact. In 2018, biallelic variants in *LZTR1* were reported to cause a recessive form of NS in 12 families (Johnston et al., 2018). The occurrence of both dominant and recessive forms of NS was confirmed the following year (Pagnamenta et al., 2019; Umeki et al., 2019). The two reports also confirmed the mutually exclusive spectrum of *LZTR1* variants occurring in dominant and recessive NS.

The leucine zipper‐like transcriptional regulator 1 (LZTR1) is a widely expressed protein that localizes to the cytoplasmic surface of the Golgi network and is characterized by six tandemly arranged Kelch motifs at the N‐terminus and two BTB/POZ (broad complex, tramtrack and bric‐a‐brac/Pox virus and zinc finger) domains at the C‐terminus (Nacak, Leptien, Fellner, Augustin, & Kroll, 2006). LZTR1 functions as a substrate receptor for cullin 3 (CUL3)‐RING ubiquitin ligase (CRL3) complexes, with the BTB domains mediating binding to CUL3 and the tandem Kelch motifs constituting the substrate recognition domain (Frattini et al., 2013). Remarkably, by using different experimental approaches, five teams provided evidence supporting a role of LZTR1 in the negative control of RAS activity and/or MAPK signaling (Abe et al., 2020; Bigenzahn et al., 2018; Castel et al., 2019; Motta et al., 2019; Steklov et al., 2018). Conflicting evidence, however, remains regarding the ability of LZTR1 to bind to multiple members of the RAS and RRAS subfamilies or specifically the RIT1 and MRAS GTPases, and whether ubiquitination of these substrates cause their degradation or subcellular redistribution.

The biochemical and functional characterization of a representative panel of missense *LZTR1* mutations associated with the dominant and recessive NS forms has enabled an understanding of their impact on intracellular signaling (Motta et al., 2019). Differently from what was observed for the recessive variants, dominantly acting, NS‐causing variants did not affect LZTR1 stability and subcellular localization nor perturb proper binding to CUL3. Moreover, overexpression of dominant, NS‐causing LZTR1 mutants, but not mutants containing missense changes implicated in recessive NS, were found to enhance stimulus‐dependent MAPK signaling. By using a homology model of the Kelch domain of LZTR1, dominantly acting *LZTR1* variants were found to map at the tips of the loops of the domain predicted to constitute the surface mediating binding to the substrate (Motta et al., 2019). Taken together, these data suggest a model that considers LZTR1 as operating in a CRL3 complex that negatively modulates RAS‐MAPK signaling. In this complex, LZTR1 functions as a substrate receptor that negatively control RAS proteins pool and their availability, either directly or indirectly. Based on this model, dominant NS‐causing *LZTR1* mutations specifically affect the surface of the Kelch domain mediating binding of the substrate to the CRL3 complex, impairing the adapter function of LZTR1, but do not perturb its binding to CUL3. As a consequence, pathogenic variants exert a dominant negative effect causing impaired substrate ubiquitination and degradation. A similar effect requires biallelic hits when variants have LoF behavior, either affecting protein synthesis, stability, or binding to CUL3 (Motta et al., 2019).

Subjects with pathogenic *LZTR1* variants generally show a “classical” NS phenotype, having short stature, typical facies, short/webbed neck, pectus deformity, cardiac involvement, and intellectual disability as recurrent features (Table 2). The most common cardiac defects include HCM, pulmonary valve stenosis (PVS), and atrial septal defect (Johnston et al., 2018; Umeki et al., 2019; Yamamoto et al., 2015; Pagnamenta et al., 2019). The available current data indicate that affected subjects with biallelic variants show a high prevalence of HCM (70%; Pagnamenta et al., 2020). Large size cohorts are required to confirm this clinically relevant genotype–phenotype correlation.

### 3.3 Loss of SPRED2 function cause a recessive form of Noonan syndrome

The three members of the SPRED family (i.e., SPRED1, SPRED2, and SPRED3) are negative regulators of signaling elicited by cell‐surface receptor tyrosine kinases. Specifically, they negatively control MAPK signaling by favoring neurofibromin binding to activated GTP‐bound RAS (Lorenzo & McCormick, 2020). Among these, SPRED3 appears to have a lower inhibitory activity (Kato et al., 2003), possibly as a result of a reduced binding affinity for neurofibromin (Hirata et al., 2016). Wide and partially overlapping expression patterns have been reported for *SPRED1* and *SPRED2*, with the former being more broadly expressed during embryogenesis (Engelhardt et al., 2004), while a more restricted expression has been reported for *SPRED3*, being limited to the brain (Kato et al., 2003). These observations suggest a differential role of the three proteins in controlling MAPK signaling, which is in line with the different phenotypes characterizing the generated knock‐out mice (Bundschu et al., 2005; Denayer et al., 2008; Inoue et al., 2005; Motta et al., 2021). Of note, partial redundancy in Spred1 and Spred2 function has been documented (Taniguchi et al., 2007).

The SPRED proteins share a similar domain organization and function (Bundschu, Walter, & Schuh, 2007; Lorenzo & McCormick, 2020). These proteins are characterized by an N‐terminal Enabled/VASP homology 1 (EVH1) domain, a central c‐Kit related binding domain (KBD; missing in SPRED3), and a C‐terminal cysteine‐rich Sprouty‐related (SPR) domain. The EVH1 domain is essential for the inhibitory function of SPRED proteins exerted on RAS since it mediates binding of the protein to neurofibromin (King et al., 2005; Yan et al., 2020). The SPR domain is subjected to post‐translational processing (i.e., palmitoylation) and is responsible for binding of SPRED proteins to the cytoplasmic leaflet of membranes (Wakioka et al., 2001; Lim et al., 2002; Kato et al., 2003).

Biallelic LoF variants in *SPRED2* (MIM: 609292) have recently been identified as underlying a disorder clinically resembling NS (Table 2; Motta et al., 2021). Four affected subjects from three unrelated families have been reported, though at least two additional individuals with similar phenotype and carrying biallelic inactivating *SPRED2* variants have been identified (Tartaglia, unpublished data). Shared features of the originally reported patients include DD/ID, facial dysmorphism (e.g., bitemporal narrowing, hypertelorism, down‐slanting palpebral fissures, low‐set/posteriorly rotated ears), low posterior hairline with a webbed/short neck, and cardiac involvement (HCM and/or PVS). Short stature with relative macrocephaly was documented in three individuals, and typical NS chest anomalies and other skeletal defects (spine) were also common findings. No lymphedema or skin/ectodermal anomalies were noted except for the occurrence of deep palmar creases (2/4 cases; Motta et al., 2021). Of note, while CALS and freckling are common in Legius syndrome, a RASopathy caused by heterozygous LoF variants in *SPRED1* (Brems & Legius, 2013), these features were not observed in subjects with biallelic LoF of *SPRED2*. The recessive inheritance of this new RASopathy further indicates a differential requirement of SPRED2 and SPRED1 function in developmental processes.

The identified pathogenic variants include missense, frameshift and truncating changes. All tested variants were demonstrated to variably affect SPRED2 stability, causing accelerated degradation, or impair proper targeting of the protein to the plasma membrane upon stimulation or protein binding to neurofibromin (Motta et al., 2021). A similar behavior had previously been reported for the *SPRED1* pathogenic variants causing LGSS (Stowe et al., 2012; Hirata et al., 2016; Yan et al., 2020). These effects converge toward a flawed down‐modulation of RAS‐MAPK signaling resulting in an overall stimulus‐dependent hyperactivation of the MAPK cascade, which was confirmed in transfected cell lines and primary fibroblasts, respectively (Motta et al., 2021).

### 3.4 Activating variants of *RRAS2* are a rare cause of Noonan syndrome

The RAS superfamily includes more than 100 small GTPases that control a wide array of cellular processes (Goitre, Trapani, Trabalzini, & Retta, 2014). RRAS, RRAS2, and MRAS are the three members of the RRAS subfamily considered as the closest relatives of the “classic” RAS proteins, HRAS, KRAS, and NRAS (Weber & Carroll, 2021). While the physiological role of these GTPases is still poorly understood, the identification of pathogenic variants in both cancer and developmental disorders document their important function on cellular processes (Flex et al., 2014; Ceremsak et al., 2016; Higgins et al., 2017; Capri et al., 2019; Niihori et al., 2019; Motta, Sagi‐Dain, et al., 2020). The functional motifs and domains of these six GTPases are relatively conserved, including the post‐translational processing at the C‐terminus, which however involves different lipidation events in individual proteins (Weber & Carroll, 2021). RRAS is geranylgeranylated and palmitoylated, whereas MRAS is only geranylgeranylated. Similar to the “classic” RAS proteins, RRAS2 is palmitoylated and farnesylated, which might explain why this GTPase strongly activates MAPK signaling compared with RRAS and MRAS (Weber & Carroll, 2021). Similarly, the regulatory mechanism controlling the switch‐on/switch‐off of these GTPases is conserved. “Classic” RAS and RRAS GTPases share some of the GEFs and GAPs regulating their activity, though specificity is also present (Weber & Carroll, 2021). RRAS proteins are able to activate redundant and distinct effectors, and variably activate the MAPK cascade by different mechanisms.

RRAS2, also known as teratocarcinoma oncogene 21 (TC21), being originally cloned from a human teratocarcinoma cDNA library (A. M. Chan, Miki, Meyers, & Aaronson, 1994), shares >50% amino acid sequence homology with HRAS, which reaches 80% when excluding the hypervariable C‐terminal tail. RRAS2 controls multiple cellular processes, including proliferation, survival, and migration, and its functional dysregulation has been documented to contribute to oncogenesis (COSMIC database, https://cancer.sanger.ac.uk/cosmic). A number of oncogenic variants have been reported in a variety of solid tumors, including carcinomas of the endometrium, prostate, lung and liver. These somatic mutations are missense and affect residues homologous to the cancer‐associated ones mutated in HRAS, KRAS and NRAS. Of note, the p.Gln72Leu change (equivalent to p.Gln61Leu in “classical” RAS proteins) was identified as driver event in isolated JMML (Ceremsak et al., 2016). Using an inducible *RRAS2* ^*Q72L*^ knock‐in mouse model, it was recently shown that this mutation triggers rapid development of a wide spectrum of tumors having limited overlap with those originated by oncogenic mutations in “classical” RAS genes, and showing tissue‐specific pharmacological vulnerabilities, which however not included inhibition of MAPK signaling (Fernandez‐Pisonero et al., 2022).

More recently, *RRAS2* was independently identified by two teams as a gene implicated in NS (Capri et al., 2019; Niihori et al., 2019). Structural inspection of the affected amino acids indicates that the identified mutations affect residues localized around the nucleotide binding pocket of the GTPase. These residues do not only play a critical role in GDP/GTP exchange and GTP hydrolysis but are also involved in stabilization of the switch regions, which mediate binding of RRAS2 to regulators and effectors. Biochemical analyses have confirmed these structural predictions, documenting an increased intrinsic and stimulated nucleotide exchange and reduced GTP hydrolysis in RRAS2^A70T^. The tested mutants were observed to function as hyperactive proteins in ELK1 transactivation experiments and variably promote increased MEK/ERK phosphorylation, while no obvious effect on the PI3K‐AKT pathway was documented (Capri et al., 2019; Niihori et al., 2019). A subset of variants was also functionally assessed in vivo using zebrafish as a model system, documenting their impact on developmental processes affected in NS (Niihori et al., 2019).

Nine patients were originally reported. The overall phenotype associated with *RRAS2* mutations fits well within the clinical spectrum of NS even though they appeared variable in terms of severity, with most subjects having features fitting typical NS (Table 2). Some patients, however, showed a relatively mild phenotype, while two had a complex and particularly severe condition and neonatal lethality (Capri et al., 2019; Niihori et al., 2019). Of note, prenatal features (nuchal edema, polyhydramnios, and/or cardiomyopathy) were reported in a large proportion of cases, and only a small proportion of the reported patients showed PVS or HCM, though these associations require larger cohorts to be validated. One additional subject carrying a de novo missense change affecting Gln^72^ (p.Gln72Leu), identified prenatally due to macrosomia, hydrocephalus, Dandy Walker malformation, and suspected arrhythmia, was more recently reported (Weinstock & Sadler, 2022). Similar to the two originally described patients heterozygous for nucleotide substitutions affecting this residue (corresponding to Gln^61^ of “classical” RAS proteins), this subject showed a severe and rapid lethal course of NS. He also was diagnosed with JMML. These findings, which provide the first evidence of the occurrence of genotype–phenotype correlations for this gene, indicate that mutations in *RRAS2* are generally associated with a classic NS phenotype (Capri et al., 2019; Niihori et al., 2019) but can be associated with a particularly severe disorder depending on the strength of the activating behavior. Based on the large screened cohorts, *RRAS2* mutations are expected to account for a small proportion of NS.

### 3.5 MRAS and the SHOC2/PPP1CB complex

One of the genes recently reported to be implicated in NS is *MRAS* (MIM: 608435), which encodes a member of the RRAS subfamily (see above). This GTPase is implicated in a wide array of cellular processes (e.g., differentiation, cytoskeletal remodeling, polarity, and cell migration) by modulating multiple signaling pathways, including the MAPK and PI3K‐AKT cascades (Young & Rodriguez‐Viciana, 2018; Endo, 2020). While MRAS is a weaker activator of the MAPK pathway compared to the members the RAS subfamily (Rodriguez‐Viciana, Sabatier, & McCormick, 2004), it is also part of a recently characterized circuit that positively regulates MAPK signaling by promoting SHOC2‐mediated translocation of the catalytic protein phosphatase 1 subunit (PP1C) to the plasma membrane. This is a key event that is required for the stable interaction of RAF proteins with GTP‐bound RAS and subsequent activation of the kinases (Rodriguez‐Viciana, Oses‐Prieto, Burlingame, Fried, & McCormick, 2006). SHOC2 is a leucine‐rich repeat‐containing protein functioning as PP1 regulatory subunit. The protein is mutated in the vast majority of patients with Mazzanti syndrome (MIM: PS607721; Cordeddu et al., 2009; Motta et al., 2022), a RASopathy closely related to NS. These patients generally share a de novo missense change (p.Ser2Gly) promoting constitutive targeting of SHOC2 to the plasma membrane due to aberrant co‐translational processing (N‐myristoylation; Cordeddu et al., 2009). The other member of the complex, PP1C, is known to interact with hundreds of regulatory proteins conferring substrate specificity and unique properties. Of note, missense mutations in *PPP1CB* [MIM: 600590], encoding one of the three catalytic PP1 subunits, account for the residual cases of Mazzanti syndrome (Gripp et al., 2016).

While activating mutations in *MRAS* are rarely found in cancer (COSMIC database), a narrow spectrum of de novo variants has recently been reported in six patients with clinical diagnosis of NS (Higgins et al., 2017; Suzuki et al., 2019; Motta, Sagi‐Dain, et al., 2020; Pires et al., 2021). The three affected residues, Gly^23^, Thr^68^, and Gln^71^, are highly conserved in all RAS orthologs and paralogs; they have a crucial role in the catalytic activity of the GTPase, and the corresponding codons (Gly^13^, Thr^58^, and Gln^61^ in H/K/NRAS) are well known hotspots for cancer‐associated and/or RASopathy‐causing mutations in other RAS proteins (Motta, Sagi‐Dain, et al., 2020). One mutation was recurrent (p.Thr68Ile) and two involved the same residue (p.Gly23Arg/Val). The predicted hyperactive behavior has experimentally been confirmed for three mutants (p.Gly23Arg, p.Gly23Val, and p.Thr68Ile), documenting impaired GTPase activity, which was associated with constitutive plasma membrane targeting, prolonged localization in non‐raft microdomains, enhanced binding to PPP1CB and SHOC2, and variably increased MAPK and PI3K‐AKT activation, which was cell context‐dependent (Motta, Sagi‐Dain, et al., 2020).

Among the cardinal features of NS, all subjects had HCM, which was particularly severe in five subjects, leading to cardiac features and neonatal death in two (Table 2; Higgins et al., 2017; Suzuki et al., 2019; Motta, Sagi‐Dain, et al., 2020; Pires et al., 2021). This finding suggests that *MRAS* mutations should be considered as a particularly high risk factor for the development of early‐onset and severe HCM (having poor prognosis in most cases), similar to what has been reported for mutations in *RIT1*, *PTPN11* and *RAF1* (limited to specific classes), and *LZTR1* (limited to biallelic events). Two large cohorts of patients were systematically assessed for *MRAS* mutations (Motta, Sagi‐Dain, et al., 2020). A single patient was identified among 288 individuals with a clinical diagnosis of a RASopathy who had previously been tested negative for all known RASopathy‐associated genes, and no mutation was identified in an unselected cohort of 1840 subjects with a clinical diagnosis of RASopathy or with features suggestive of these disorders. These data suggest that the frequency of MRAS‐related NS is relatively rare, possibly due to the severe, often neonatally lethal phenotype.

The finding that the clinical features associated with *MRAS* mutations fall well within the NS phenotypic spectrum (see below) and are not reminiscent of Mazzanti syndrome suggests that the impact of dysregulated MRAS function on intracellular signaling is not equivalent to the dysregulation driven by upregulated SHOC2 and PPP1CB function. Another aspect requiring further investigation is the specific link between MRAS hyperactivation and HCM. Accumulated evidence indicates that both the MAPK and PI3K‐AKT–mTOR pathways contribute to HCM in RASopathies (Gelb, Roberts, & Tartaglia, 2015), and depending upon the cellular context, NS‐causing MRAS mutants differentially impact MAPK and PI3K‐AKT signaling cascades (Motta, Sagi‐Dain, et al., 2020). Hyperactive MRAS was demonstrated to be both necessary and sufficient to elicit a cardiac hypertrophy phenotype in iPSC‐derived cardiomyocytes (Higgins, Bos, Dotzler, John Kim, & Ackerman, 2019). While RAS‐MAPK signaling was confirmed to be upregulated and contribute to the hypertrophic endophenotype in the used in vitro model, further effort is required to a deeper understanding of the pathway(s) implicated in *MRAS* variant‐related HCM.

## 4 CLOSING REMARKS

While “functional candidacy” has successfully been used to identify several genes implicated in RASopathies, more recently the use of a “hypothesis‐free” approach based on genome sequencing has allowed to discover novel disease genes whose function in RAS signaling had not been appreciated or was poorly defined. Notwithstanding these successes, the clinical diagnosis remains molecularly unsubstantiated in a significant proportion of individuals (up to 20% of cases with suggestive phenotypes). This indicates that other genes are implicated in this family of disorders or that genocopies (i.e., diseases with clinical overlap with RASopathies but with different pathogenetic bases) account for these patients. We anticipate that the routine application of genomic sequencing in the clinical practice will enable answering this open question in the next few years and is expected to provide further insights on other molecular mechanisms and circuits functionally linked to RAS signaling.

## CONFLICT OF INTEREST

None.

## Data Availability

Data sharing not applicable to this article as no datasets were generated or analysed during the current study.

## References

[ajmgc32012-bib-0001] Abe, T. , Umeki, I. , Kanno, S. I. , Inoue, S. I. , Niihori, T. , & Aoki, Y. (2020). LZTR1 facilitates polyubiquitination and degradation of RAS‐GTPases. Cell Death and Differentiation, 27(3), 1023–1035. 10.1038/s41418-019-0395-5 31337872 PMC7206011

[ajmgc32012-bib-0002] Aoki, Y. , & Matsubara, Y. (2013). Ras/MAPK syndromes and childhood hemato‐oncological diseases. International Journal of Hematology, 97(1), 30–36. 10.1007/s12185-012-1239-y 23250860

[ajmgc32012-bib-0003] Aoki, Y. , Niihori, T. , Banjo, T. , Okamoto, N. , Mizuno, S. , Kurosawa, K. , … Matsubara, Y. (2013). Gain‐of‐function mutations in RIT1 cause Noonan syndrome, a RAS/MAPK pathway syndrome. American Journal of Human Genetics, 93(1), 173–180. 10.1016/j.ajhg.2013.05.021 23791108 PMC3710767

[ajmgc32012-bib-0004] Aoki, Y. , Niihori, T. , Inoue, S. , & Matsubara, Y. (2016). Recent advances in RASopathies. Journal of Human Genetics, 61(1), 33–39. 10.1038/jhg.2015.114 26446362

[ajmgc32012-bib-0005] Aoki, Y. , Niihori, T. , Kawame, H. , Kurosawa, K. , Ohashi, H. , Tanaka, Y. , … Matsubara, Y. (2005). Germline mutations in HRAS proto‐oncogene cause Costello syndrome. Nature Genetics, 37(10), 1038–1040. 10.1038/ng1641 16170316

[ajmgc32012-bib-0006] Bader‐Meunier, B. , Tchernia, G. , Miélot, F. , Fontaine, J. L. , Thomas, C. , Lyonnet, S. , … Dommergues, J. P. (1997). Occurrence of myeloproliferative disorder in patients with Noonan syndrome. The Journal of Pediatrics, 130(6), 885–889. 10.1016/s0022-3476(97)70273-7 9202609

[ajmgc32012-bib-0007] Bentires‐Alj, M. , Paez, J. G. , David, F. S. , Keilhack, H. , Halmos, B. , Naoki, K. , … Neel, B. G. (2004). Activating mutations of the Noonan syndrome‐associated SHP2/PTPN11 gene in human solid tumors and adult acute myelogenous leukemia. Cancer Research, 64(24), 8816–8820. 10.1158/0008-5472.CAN-04-1923 15604238

[ajmgc32012-bib-0008] Bergoug, M. , Doudeau, M. , Godin, F. , Mosrin, C. , Vallée, B. , & Bénédetti, H. (2020). Neurofibromin structure, functions and regulation. Cell, 9(11), 2365. 10.3390/cells9112365 PMC7692384 33121128

[ajmgc32012-bib-0009] Bigenzahn, J. W. , Collu, G. M. , Kartnig, F. , Pieraks, M. , Vladimer, G. I. , Heinz, L. X. , … Superti‐Furga, G. (2018). LZTR1 is a regulator of RAS ubiquitination and signaling. Science, 362(6419), 1171–1177. 10.1126/science.aap8210 30442766 PMC6794158

[ajmgc32012-bib-0010] Brems, H. , Chmara, M. , Sahbatou, M. , Denayer, E. , Taniguchi, K. , Kato, R. , … Legius, E. (2007). Germline loss‐of‐function mutations in SPRED1 cause a neurofibromatosis 1‐like phenotype. Nature Genetics, 39(9), 1120–1126. 10.1038/ng2113 17704776

[ajmgc32012-bib-0011] Brems, H. , & Legius, E. (2013). Legius syndrome, an update. Molecular pathology of mutations in SPRED1. The Keio Journal of Medicine, 62(4), 107–112. 10.2302/kjm.2013-0002-re 24334617

[ajmgc32012-bib-0012] Brenan, L. , Andreev, A. , Cohen, O. , Pantel, S. , Kamburov, A. , Cacchiarelli, D. , … Johannessen, C. M. (2016). Phenotypic characterization of a comprehensive set of MAPK1/ERK2 missense mutants. Cell Reports, 17(4), 1171–1183. 10.1016/j.celrep.2016.09.061 27760319 PMC5120861

[ajmgc32012-bib-0013] Bundschu, K. , Knobeloch, K. P. , Ullrich, M. , Schinke, T. , Amling, M. , Engelhardt, C. M. , … Schuh, K. (2005). Gene disruption of Spred‐2 causes dwarfism. The Journal of Biological Chemistry, 280(31), 28572–28580. 10.1074/jbc.M503640200 15946934

[ajmgc32012-bib-0014] Bundschu, K. , Walter, U. , & Schuh, K. (2007). Getting a first clue about SPRED functions. BioEssays, 29(9), 897–907. 10.1002/bies.20632 17691106

[ajmgc32012-bib-0015] Canagarajah, B. J. , Khokhlatchev, A. , Cobb, M. H. , & Goldsmith, E. J. (1997). Activation mechanism of the MAP kinase ERK2 by dual phosphorylation. Cell, 90, 859–869. 10.1016/s0092-8674(00)80351-7 9298898

[ajmgc32012-bib-0016] Capri, Y. , Flex, E. , Krumbach, O. H. F. , Carpentieri, G. , Cecchetti, S. , Lißewski, C. , … Zenker, M. (2019). Activating mutations of RRAS2 are a rare cause of Noonan syndrome. American Journal of Human Genetics, 104(6), 1223–1232. 10.1016/j.ajhg.2019.04.013 31130282 PMC6562003

[ajmgc32012-bib-0017] Carta, C. , Pantaleoni, F. , Bocchinfuso, G. , Stella, L. , Vasta, I. , Sarkozy, A. , … Tartaglia, M. (2006). Germline missense mutations affecting KRAS isoform B are associated with a severe Noonan syndrome phenotype. American Journal of Human Genetics, 79(1), 129–135. 10.1086/504394 16773572 PMC1474118

[ajmgc32012-bib-0018] Castel, P. , Cheng, A. , Cuevas‐Navarro, A. , Everman, D. B. , Papageorge, A. G. , Simanshu, D. K. , … McCormick, F. (2019). RIT1 oncoproteins escape LZTR1‐mediated proteolysis. Science, 363(6432), 1226–1230. 10.1126/science.aav1444 30872527 PMC6986682

[ajmgc32012-bib-0019] Ceremsak, J. J. , Yu, A. , Esquivel, E. , Lissewski, C. , Zenker, M. , Loh, M. L. , & Stieglitz, E. (2016). Germline RRAS2 mutations are not associated with Noonan syndrome. Journal of Medical Genetics, 53(11), 728. 10.1136/jmedgenet-2016-103889 27055474

[ajmgc32012-bib-0020] Chan, A. M. , Miki, T. , Meyers, K. A. , & Aaronson, S. A. (1994). A human oncogene of the RAS superfamily unmasked by expression cDNA cloning. Proceedings of the National Academy of Sciences of the United States of America, 91(16), 7558–7562. 10.1073/pnas.91.16.7558 8052619 PMC44441

[ajmgc32012-bib-0021] Chan, R. J. , Leedy, M. B. , Munugalavadla, V. , Voorhorst, C. S. , Li, Y. , Yu, M. , & Kapur, R. (2005). Human somatic PTPN11 mutations induce hematopoietic‐cell hypersensitivity to granulocyte‐macrophage colony‐stimulating factor. Blood, 105(9), 3737–3742. 10.1182/blood-2004-10-4002 15644411 PMC1895012

[ajmgc32012-bib-0022] Chen, H. , Li, X. , Liu, X. , Wang, J. , Zhang, Z. , Wu, J. , … Fu, L. (2019). Clinical and mutation profile of pediatric patients with RASopathy‐associated hypertrophic cardiomyopathy: Results from a Chinese cohort. Orphanet Journal of Rare Diseases, 14(1), 29. 10.1186/s13023-019-1010-z 30732632 PMC6367752

[ajmgc32012-bib-0023] Chen, P. C. , Yin, J. , Yu, H. W. , Yuan, T. , Fernandez, M. , Yung, C. K. , … Kucherlapati, R. (2014). Next‐generation sequencing identifies rare variants associated with Noonan syndrome. Proceedings of the National Academy of Sciences of the United States of America, 111(31), 11473–11478. 10.1073/pnas.1324128111 25049390 PMC4128129

[ajmgc32012-bib-0024] Choong, K. , Freedman, M. H. , Chitayat, D. , Kelly, E. N. , Taylor, G. , & Zipursky, A. (1999). Juvenile myelomonocytic leukemia and Noonan syndrome. Journal of Pediatric Hematology/Oncology, 21(6), 523–527 PMID: 10598665. 10598665

[ajmgc32012-bib-0025] Cirstea, I. C. , Kutsche, K. , Dvorsky, R. , Gremer, L. , Carta, C. , Horn, D. , … Zenker, M. (2010). A restricted spectrum of NRAS mutations causes Noonan syndrome. Nature Genetics, 42(1), 27–29. 10.1038/ng.497 19966803 PMC3118669

[ajmgc32012-bib-0026] Cordeddu, V. , Di Schiavi, E. , Pennacchio, L. A. , Ma'ayan, A. , Sarkozy, A. , Fodale, V. , … Tartaglia, M. (2009). Mutation of SHOC2 promotes aberrant protein N‐myristoylation and causes Noonan‐like syndrome with loose anagen hair. Nature Genetics, 41(9), 1022–1026. 10.1038/ng.425 19684605 PMC2765465

[ajmgc32012-bib-0027] Cordeddu, V. , Yin, J. C. , Gunnarsson, C. , Virtanen, C. , Drunat, S. , Lepri, F. , … Tartaglia, M. (2015). Activating mutations affecting the Dbl homology domain of SOS2 cause Noonan syndrome. Human Mutation, 36(11), 1080–1087. 10.1002/humu.22834 26173643 PMC4604019

[ajmgc32012-bib-0028] Cox, A. D. , Fesik, S. W. , Kimmelman, A. C. , Luo, J. , & Der, C. J. (2014). Drugging the undruggable RAS: Mission possible? Nature Reviews. Drug Discovery, 13(11), 828–851. 10.1038/nrd4389 25323927 PMC4355017

[ajmgc32012-bib-0029] Denayer, E. , Ahmed, T. , Brems, H. , Van Woerden, G. , Borgesius, N. Z. , Callaerts‐Vegh, Z. , … Balschun, D. (2008). Spred1 is required for synaptic plasticity and hippocampus‐dependent learning. The Journal of Neuroscience, 28(53), 4443–14449. 10.1523/JNEUROSCI.4698-08.2008 PMC6671253 19118178

[ajmgc32012-bib-0030] Digilio, M. C. , Conti, E. , Sarkozy, A. , Mingarelli, R. , Dottorini, T. , Marino, B. , … Dallapiccola, B. (2002). Grouping of multiple‐lentigines/LEOPARD and Noonan syndromes on the PTPN11 gene. American Journal of Human Genetics, 71(2), 389–394. 10.1086/341528 12058348 PMC379170

[ajmgc32012-bib-0031] Endo, T. (2020). M‐Ras is muscle‐Ras, moderate‐Ras, mineral‐Ras, migration‐Ras, and many more‐Ras. Experimental Cell Research, 397(1), 112342. 10.1016/j.yexcr.2020.112342 33130177

[ajmgc32012-bib-0032] Engelhardt, C. M. , Bundschu, K. , Messerschmitt, M. , Renné, T. , Walter, U. , Reinhard, M. , & Schuh, K. (2004). Expression and subcellular localization of Spred proteins in mouse and human tissues. Histochemistry and Cell Biology, 122(6), 527–538. 10.1007/s00418-004-0725-6 15580519

[ajmgc32012-bib-0033] Farncombe, K. M. , Thain, E. , Barnett‐Tapia, C. , Sadeghian, H. , & Kim, R. H. (2022). LZTR1 molecular genetic overlap with clinical implications for Noonan syndrome and schwannomatosis. BMC Medical Genomics, 15(1), 160. 10.1186/s12920-022-01304-x 35840934 PMC9288044

[ajmgc32012-bib-0034] Fernandez‐Pisonero, I. , Clavaín, L. , Robles‐Valero, J. , Lorenzo‐Martín, L. F. , Caloto, R. , Nieto, B. , … Bustelo, X. R. (2022). A hotspot mutation targeting the R‐RAS2 GTPase acts as a potent oncogenic driver in a wide spectrum of tumors. Cell Reports, 38(11), 110522. 10.1016/j.celrep.2022.110522 35294890

[ajmgc32012-bib-0035] Flex, E. , Jaiswal, M. , Pantaleoni, F. , Martinelli, S. , Strullu, M. , Fansa, E. K. , … Tartaglia, M. (2014). Activating mutations in RRAS underlie a phenotype within the RASopathy spectrum and contribute to leukaemogenesis. Human Molecular Genetics, 23(16), 4315–4327. 10.1093/hmg/ddu148 24705357 PMC4103678

[ajmgc32012-bib-0036] Fragale, A. , Tartaglia, M. , Wu, J. , & Gelb, B. D. (2004). Noonan syndrome‐associated SHP2/PTPN11 mutants cause EGF‐dependent prolonged GAB1 binding and sustained ERK2/MAPK1 activation. Human Mutation, 23(3), 267–277. 10.1002/humu.20005 14974085

[ajmgc32012-bib-0037] Frattini, V. , Trifonov, V. , Chan, J. M. , Castano, A. , Lia, M. , Abate, F. , … Iavarone, A. (2013). The integrated landscape of driver genomic alterations in glioblastoma. Nature Genetics, 45(10), 1141–1149. 10.1038/ng.2734 23917401 PMC3799953

[ajmgc32012-bib-0038] Gelb, B. D. , Roberts, A. E. , & Tartaglia, M. (2015). Cardiomyopathies in Noonan syndrome and the other RASopathies. Progress in Pediatric Cardiology, 39(1), 13–19. 10.1016/j.ppedcard.2015.01.002 26380542 PMC4568836

[ajmgc32012-bib-0039] Ghose, R. (2019). Nature of the pre‐chemistry ensemble in mitogen‐activated protein kinases. Journal of Molecular Biology, 431(2), 145–157. 10.1016/j.jmb.2018.12.007 30562484

[ajmgc32012-bib-0040] Goitre, L. , Trapani, E. , Trabalzini, L. , & Retta, S. F. (2014). The Ras superfamily of small GTPases: The unlocked secrets. Methods in Molecular Biology, 1120, 1–18. 10.1007/978-1-62703-791-4_1 24470015

[ajmgc32012-bib-0041] Grant, A. R. , Cushman, B. J. , Cavé, H. , Dillon, M. W. , Gelb, B. D. , Gripp, K. W. , … Zenker, M. (2018). Assessing the gene‐disease association of 19 genes with the RASopathies using the ClinGen gene curation framework. Human Mutation, 39(11), 1485–1493. 10.1002/humu.23624 30311384 PMC6326381

[ajmgc32012-bib-0042] Gripp, K. W. , Aldinger, K. A. , Bennett, J. T. , Baker, L. , Tusi, J. , Powell‐Hamilton, N. , … Dobyns, W. B. (2016). A novel rasopathy caused by recurrent de novo missense mutations in PPP1CB closely resembles Noonan syndrome with loose anagen hair. American Journal of Medical Genetics. Part A, 170(9), 2237–2247. 10.1002/ajmg.a.37781 27264673 PMC5134331

[ajmgc32012-bib-0043] Güemes, M. , Martín‐Rivada, Á. , Ortiz‐Cabrera, N. V. , Martos‐Moreno, G. Á. , Pozo‐Román, J. , & Argente, J. (2019). LZTR1: Genotype expansion in Noonan syndrome. Hormone Research in Pædiatrics, 92(4), 269–275. 10.1159/000502741 31533111

[ajmgc32012-bib-0044] Hanna, N. , Montagner, A. , Lee, W. H. , Miteva, M. , Vidal, M. , Vidaud, M. , … Raynal, P. (2006). Reduced phosphatase activity of SHP‐2 in LEOPARD syndrome: Consequences for PI3K binding on Gab1. FEBS Letters, 580(10), 2477–2482. 10.1016/j.febslet.2006.03.088 16638574

[ajmgc32012-bib-0045] Higgins, E. M. , Bos, J. M. , Dotzler, S. M. , John Kim, C. S. , & Ackerman, M. J. (2019). MRAS variants cause cardiomyocyte hypertrophy in patient‐specific induced pluripotent stem cell‐derived cardiomyocytes: Additional evidence for MRAS as a definitive Noonan syndrome‐susceptibility gene. Circulation: Genomic and Precision Medicine, 12(11), e002648. 10.1161/CIRCGEN.119.002648 31638832

[ajmgc32012-bib-0046] Higgins, E. M. , Bos, J. M. , Mason‐Suares, H. , Tester, D. J. , Ackerman, J. P. , MacRae, C. A. , … Ackerman, M. J. (2017). Elucidation of MRAS‐mediated Noonan syndrome with cardiac hypertrophy. JCI Insight, 2(5), e91225. 10.1172/jci.insight.91225 28289718 PMC5333962

[ajmgc32012-bib-0047] Hirata, Y. , Brems, H. , Suzuki, M. , Kanamori, M. , Okada, M. , Morita, R. , … Yoshimura, A. (2016). Interaction between a domain of the negative regulator of the Ras‐ERK pathway, SPRED1 protein, and the GTPase‐activating protein‐related domain of neurofibromin is implicated in Legius syndrome and neurofibromatosis type 1. The Journal of Biological Chemistry, 291(7), 3124–3134. 10.1074/jbc.M115.703710 26635368 PMC4751360

[ajmgc32012-bib-0048] Hugues, L. , Cavé, H. , Philippe, N. , Pereira, S. , Fenaux, P. , & Preudhomme, C. (2005). Mutations of PTPN11 are rare in adult myeloid malignancies. Haematologica, 90(6), 853–854 PMID: 15951301. 15951301

[ajmgc32012-bib-0049] Inoue, H. , Kato, R. , Fukuyama, S. , Nonami, A. , Taniguchi, K. , Matsumoto, K. , … Yoshimura, A. (2005). Spred‐1 negatively regulates allergen‐induced airway eosinophilia and hyperresponsiveness. The Journal of Experimental Medicine, 201(1), 73–82. 10.1084/jem.20040616 15630138 PMC2212755

[ajmgc32012-bib-0050] Jacquinet, A. , Bonnard, A. , Capri, Y. , Martin, D. , Sadzot, B. , Bianchi, E. , … Verloes, A. (2020). Oligo‐astrocytoma in LZTR1‐related Noonan syndrome. European Journal of Medical Genetics, 63(1), 103617. 10.1016/j.ejmg.2019.01.007 30664951

[ajmgc32012-bib-0051] Johan, M. F. , Bowen, D. T. , Frew, M. E. , Goodeve, A. C. , Wilson, G. A. , Peake, I. R. , & Reilly, J. T. (2004). Mutations in PTPN11 are uncommon in adult myelodysplastic syndromes and acute myeloid leukaemia. British Journal of Haematology, 124(6), 843–844. 10.1111/j.1365-2141.2004.04862.x 15009076

[ajmgc32012-bib-0052] Johnston, J. J. , van der Smagt, J. J. , Rosenfeld, J. A. , Pagnamenta, A. T. , Alswaid, A. , Baker, E. H. , … Biesecker, L. G. (2018). Autosomal recessive Noonan syndrome associated with biallelic LZTR1 variants. Genetics in Medicine, 20(10), 1175–1185. 10.1038/gim.2017.249 29469822 PMC6105555

[ajmgc32012-bib-0053] Kato, R. , Nonami, A. , Taketomi, T. , Wakioka, T. , Kuroiwa, A. , Matsuda, Y. , & Yoshimura, A. (2003). Molecular cloning of mammalian Spred‐3 which suppresses tyrosine kinase‐mediated Erk activation. Biochemical and Biophysical Research Communications, 302(4), 767–772. 10.1016/s0006-291x(03)00259-6 12646235

[ajmgc32012-bib-0054] Keilhack, H. , David, F. S. , McGregor, M. , Cantley, L. C. , & Neel, B. G. (2005). Diverse biochemical properties of Shp2 mutants. Implications for disease phenotypes. The Journal of Biological Chemistry, 280(35), 30984–30993. 10.1074/jbc.M504699200 15987685

[ajmgc32012-bib-0055] King, J. A. , Straffon, A. F. , D'Abaco, G. M. , Poon, C. L. , Stacey, S. T. I. , Smith, C. M. , … Hovens, C. M. (2005). Distinct requirements for the Sprouty domain for functional activity of Spred proteins. The Biochemical Journal, 388(Pt 2), 445–454. 10.1042/BJ20041284 15683364 PMC1138951

[ajmgc32012-bib-0056] Klomp, J. E. , Klomp, J. A. , & Der, C. J. (2021). The ERK mitogen‐activated protein kinase signaling network: The final frontier in RAS signal transduction. Biochemical Society Transactions, 49(1), 253–267. 10.1042/BST20200507 33544118 PMC12691160

[ajmgc32012-bib-0057] Kolch, W. (2005). Coordinating ERK/MAPK signaling through scaffolds and inhibitors. Nature Reviews. Molecular Cell Biology, 6(11), 827–837. 10.1038/nrm1743 16227978

[ajmgc32012-bib-0058] Lake, D. , Corrêa, S. A. , & Müller, J. (2016). Negative feedback regulation of the ERK1/2 MAPK pathway. Cellular and Molecular Life Sciences, 73(23), 4397–4413. 10.1007/s00018-016-2297-8 27342992 PMC5075022

[ajmgc32012-bib-0059] Legius, E. , Schrander‐Stumpel, C. , Schollen, E. , Pulles‐Heintzberger, C. , Gewillig, M. , & Fryns, J. P. (2002). PTPN11 mutations in LEOPARD syndrome. Journal of Medical Genetics, 39(8), 571–574. 10.1136/jmg.39.8.571 12161596 PMC1735195

[ajmgc32012-bib-0060] Lim, J. , Yusoff, P. , Wong, E. S. , Chandramouli, S. , Lao, D. H. , Fong, C. W. , & Guy, G. R. (2002). The cysteine‐rich sprout translocation domain targets mitogen‐activated protein kinase inhibitory proteins to phosphatidylinositol 4,5‐bisphosphate in plasma membranes. Molecular and Cellular Biology, 22(22), 7953–7966. 10.1128/MCB.22.22.7953-7966.2002 12391162 PMC134720

[ajmgc32012-bib-0061] Loh, M. L. , Martinelli, S. , Cordeddu, V. , Reynolds, M. G. , Vattikuti, S. , Lee, C. M. , … Tartaglia, M. (2005). Acquired PTPN11 mutations occur rarely in adult patients with myelodysplastic syndromes and chronic myelomonocytic leukemia. Leukemia Research, 29(4), 459–462. 10.1016/j.leukres.2004.10.001 15725481

[ajmgc32012-bib-0062] Loh, M. L. , Vattikuti, S. , Schubbert, S. , Reynolds, M. G. , Carlson, E. , Lieuw, K. H. , … Shannon, K. M. (2004). Mutations in PTPN11 implicate the SHP‐2 phosphatase in leukemogenesis. Blood, 103(6), 2325–2331. 10.1182/blood-2003-09-3287 14644997

[ajmgc32012-bib-0063] Lorenzo, C. , & McCormick, F. (2020). SPRED proteins and their roles in signal transduction, development, and malignancy. Genes & Development, 34(21–22), 1410–1421. 10.1101/gad.341222.120 33872193 PMC7608746

[ajmgc32012-bib-0064] Martinelli, S. , De Luca, A. , Stellacci, E. , Rossi, C. , Checquolo, S. , Lepri, F. , … Tartaglia, M. (2010). Heterozygous germline mutations in the CBL tumor‐suppressor gene cause a Noonan syndrome‐like phenotype. American Journal of Human Genetics, 87(2), 250–257. 10.1016/j.ajhg.2010.06.015 20619386 PMC2917705

[ajmgc32012-bib-0065] Motta, M. , Fasano, G. , Gredy, S. , Brinkmann, J. , Bonnard, A. A. , Simsek‐Kiper, P. O. , … Tartaglia, M. (2021). SPRED2 loss‐of‐function causes a recessive Noonan syndrome‐like phenotype. American Journal of Human Genetics, 108(11), 2112–2129. 10.1016/j.ajhg.2021.09.007 34626534 PMC8595899

[ajmgc32012-bib-0066] Motta, M. , Fidan, M. , Bellacchio, E. , Pantaleoni, F. , Schneider‐Heieck, K. , Coppola, S. , … Tartaglia, M. (2019). Dominant Noonan syndrome‐causing LZTR1 mutations specifically affect the Kelch domain substrate‐recognition surface and enhance RAS‐MAPK signaling. Human Molecular Genetics, 28(6), 1007–1022. 10.1093/hmg/ddy412 30481304

[ajmgc32012-bib-0067] Motta, M. , Pannone, L. , Pantaleoni, F. , Bocchinfuso, G. , Radio, F. C. , Cecchetti, S. , … Tartaglia, M. (2020). Enhanced MAPK1 function causes a neurodevelopmental disorder within the RASopathy clinical Spectrum. American Journal of Human Genetics, 107(3), 499–513. 10.1016/j.ajhg.2020.06.018 32721402 PMC7477014

[ajmgc32012-bib-0068] Motta, M. , Sagi‐Dain, L. , Krumbach, O. H. F. , Hahn, A. , Peleg, A. , German, A. , … Zenker, M. (2020). Activating MRAS mutations cause Noonan syndrome associated with hypertrophic cardiomyopathy. Human Molecular Genetics, 29(11), 1772–1783. 10.1093/hmg/ddz108 31108500

[ajmgc32012-bib-0069] Motta, M. , Solman, M. , Bonnard, A. A. , Kuechler, A. , Pantaleoni, F. , Priolo, M. , … Tartaglia, M. (2022). Expanding the molecular spectrum of pathogenic SHOC2 variants underlying Mazzanti syndrome. Human Molecular Genetics, 31(16), 2766–2778. 10.1093/hmg/ddac071 35348676 PMC9402240

[ajmgc32012-bib-0070] Nacak, T. G. , Leptien, K. , Fellner, D. , Augustin, H. G. , & Kroll, J. (2006). The BTB‐kelch protein LZTR‐1 is a novel Golgi protein that is degraded upon induction of apoptosis. The Journal of Biological Chemistry, 281(8), 5065–5071. 10.1074/jbc.M509073200 16356934

[ajmgc32012-bib-0071] Nakagama, Y. , Takeda, N. , Ogawa, S. , Takeda, H. , Furutani, Y. , Nakanishi, T. , … Inuzuka, R. (2020). Noonan syndrome‐associated biallelic LZTR1 mutations cause cardiac hypertrophy and vascular malformations in zebrafish. Molecular Genetics & Genomic Medicine, 8(3), e1107. 10.1002/mgg3.1107 31883238 PMC7057116

[ajmgc32012-bib-0072] Nava, C. , Hanna, N. , Michot, C. , Pereira, S. , Pouvreau, N. , Niihori, T. , … Cavé, H. (2007). Cardio‐facio‐cutaneous and Noonan syndromes due to mutations in the RAS/MAPK signalling pathway: Genotype‐phenotype relationships and overlap with Costello syndrome. Journal of Medical Genetics, 44(12), 763–771. 10.1136/jmg.2007.050450 17704260 PMC2652823

[ajmgc32012-bib-0073] Niemeyer, C. M. , Kang, M. W. , Shin, D. H. , Furlan, I. , Erlacher, M. , Bunin, N. J. , … Loh, M. L. (2010). Germline CBL mutations cause developmental abnormalities and predispose to juvenile myelomonocytic leukemia. Nature Genetics, 42(9), 794–800. 10.1038/ng.641 20694012 PMC4297285

[ajmgc32012-bib-0074] Niihori, T. , Aoki, Y. , Narumi, Y. , Neri, G. , Cavé, H. , Verloes, A. , … Matsubara, Y. (2006). Germline KRAS and BRAF mutations in cardio‐facio‐cutaneous syndrome. Nature Genetics, 38(3), 294–296. 10.1038/ng1749 16474404

[ajmgc32012-bib-0075] Niihori, T. , Nagai, K. , Fujita, A. , Ohashi, H. , Okamoto, N. , Okada, S. , … Aoki, Y. (2019). Germline‐activating RRAS2 mutations cause Noonan syndrome. American Journal of Human Genetics, 104(6), 1233–1240. 10.1016/j.ajhg.2019.04.014 31130285 PMC6562005

[ajmgc32012-bib-0076] Pagnamenta, A. T. , Kaisaki, P. J. , Bennett, F. , Burkitt‐Wright, E. , Martin, H. C. , Ferla, M. P. , … Stewart, H. (2019). Delineation of dominant and recessive forms of LZTR1‐associated Noonan syndrome. Clinical Genetics, 95(6), 693–703. 10.1111/cge.13533 30859559 PMC6563422

[ajmgc32012-bib-0077] Pandit, B. , Sarkozy, A. , Pennacchio, L. A. , Carta, C. , Oishi, K. , Martinelli, S. , … Gelb, B. D. (2007). Gain‐of‐function RAF1 mutations cause Noonan and LEOPARD syndromes with hypertrophic cardiomyopathy. Nature Genetics, 39(8), 1007–1012. 10.1038/ng2073 17603483

[ajmgc32012-bib-0078] Pérez, B. , Mechinaud, F. , Galambrun, C. , Ben Romdhane, N. , Isidor, B. , Philip, N. , … Cavé, H. (2010). Germline mutations of the CBL gene define a new genetic syndrome with predisposition to juvenile myelomonocytic leukaemia. Journal of Medical Genetics, 47(10), 686–691. 10.1136/jmg.2010.076836 20543203

[ajmgc32012-bib-0079] Perin, F. , Trujillo‐Quintero, J. P. , Jimenez‐Jaimez, J. , Rodríguez‐Vázquez Del Rey, M. D. M. , Monserrat, L. , & Tercedor, L. (2019). Two novel cases of autosomal recessive Noonan syndrome associated with LZTR1 variants. Revista Española de Cardiología, 72(11), 978–980. 10.1016/j.rec.2019.05.002 31182298

[ajmgc32012-bib-0080] Peti, W. , & Page, R. (2013). Molecular basis of MAP kinase regulation. Protein Science, 22(12), 1698–1710. 10.1002/pro.2374 24115095 PMC3843625

[ajmgc32012-bib-0081] Piotrowski, A. , Xie, J. , Liu, Y. F. , Poplawski, A. B. , Gomes, A. R. , Madanecki, P. , … Messiaen, L. M. (2014). Germline loss‐of‐function mutations in LZTR1 predispose to an inherited disorder of multiple schwannomas. Nature Genetics, 46(2), 182–187. 10.1038/ng.2855 24362817 PMC4352302

[ajmgc32012-bib-0082] Pires, L. V. L. , Bordim, R. A. , Maciel, M. B. R. , Tanaka, A. C. S. , Yamamoto, G. L. , Honjo, R. S. , … Bertola, D. R. (2021). Atypical, severe hypertrophic cardiomyopathy in a newborn presenting Noonan syndrome harboring a recurrent heterozygous MRAS variant. American Journal of Medical Genetics. Part A, 185(10), 3099–3103. 10.1002/ajmg.a.62376 34080768

[ajmgc32012-bib-0083] Pylayeva‐Gupta, Y. , Grabocka, E. , & Bar‐Sagi, D. (2011). RAS oncogenes: Weaving a tumorigenic web. Nature Reviews. Cancer, 11(11), 761–774. 10.1038/nrc3106 21993244 PMC3632399

[ajmgc32012-bib-0084] Rauen, K. A. (2013). The RASopathies. Annual Review of Genomics and Human Genetics, 14, 355–369. 10.1146/annurev-genom-091212-153523 PMC4115674 23875798

[ajmgc32012-bib-0085] Razzaque, M. A. , Nishizawa, T. , Komoike, Y. , Yagi, H. , Furutani, M. , Amo, R. , … Matsuoka, R. (2007). Germline gain‐of‐function mutations in RAF1 cause Noonan syndrome. Nature Genetics, 39(8), 1013–1017. 10.1038/ng2078 17603482

[ajmgc32012-bib-0086] Reményi, A. , Good, M. C. , & Lim, W. A. (2006). Docking interactions in protein kinase and phosphatase networks. Current Opinion in Structural Biology, 16(6), 676–685. 10.1016/j.sbi.2006.10.008 17079133

[ajmgc32012-bib-0087] Roberts, A. E. , Araki, T. , Swanson, K. D. , Montgomery, K. T. , Schiripo, T. A. , Joshi, V. A. , … Kucherlapati, R. S. (2007). Germline gain‐of‐function mutations in SOS1 cause Noonan syndrome. Nature Genetics, 39(1), 70–74. 10.1038/ng1926 17143285

[ajmgc32012-bib-0088] Rodriguez‐Viciana, P. , Oses‐Prieto, J. , Burlingame, A. , Fried, M. , & McCormick, F. (2006). A phosphatase holoenzyme comprised of Shoc2/Sur8 and the catalytic subunit of PP1 functions as an M‐Ras effector to modulate Raf activity. Molecular Cell, 22(2), 217–230. 10.1016/j.molcel.2006.03.027 16630891

[ajmgc32012-bib-0089] Rodriguez‐Viciana, P. , Sabatier, C. , & McCormick, F. (2004). Signaling specificity by Ras family GTPases is determined by the full spectrum of effectors they regulate. Molecular and Cellular Biology, 24(11), 4943–4954. 10.1128/MCB.24.11.4943-4954.2004 15143186 PMC416418

[ajmgc32012-bib-0090] Rodriguez‐Viciana, P. , Tetsu, O. , Tidyman, W. E. , Estep, A. L. , Conger, B. A. , Cruz, M. S. , … Rauen, K. A. (2006). Germline mutations in genes within the MAPK pathway cause cardio‐facio‐cutaneous syndrome. Science, 311(5765), 1287–1290. 10.1126/science.1124642 16439621

[ajmgc32012-bib-0091] Roskoski, R., Jr. (2012). ERK1/2 MAP kinases: Structure, function, and regulation. Pharmacological Research, 66(2), 105–143. 10.1016/j.phrs.2012.04.005 22569528

[ajmgc32012-bib-0092] Roskoski, R., Jr. (2015). A historical overview of protein kinases and their targeted small molecule inhibitors. Pharmacological Research, 100, 1–23. 10.1016/j.phrs.2015.07.010 26207888

[ajmgc32012-bib-0093] Rubinfeld, H. , & Seger, R. (2005). The ERK cascade: A prototype of MAPK signaling. Molecular Biotechnology, 31(2), 151–174. 10.1385/MB:31:2:151 16170216

[ajmgc32012-bib-0094] Sarkozy, A. , Carta, C. , Moretti, S. , Zampino, G. , Digilio, M. C. , Pantaleoni, F. , … Tartaglia, M. (2009). Germline BRAF mutations in Noonan, LEOPARD, and cardiofaciocutaneous syndromes: Molecular diversity and associated phenotypic spectrum. Human Mutation, 30(4), 695–702. 10.1002/humu.20955 19206169 PMC4028130

[ajmgc32012-bib-0095] Schubbert, S. , Lieuw, K. , Rowe, S. L. , Lee, C. M. , Li, X. , Loh, M. L. , … Shannon, K. M. (2005). Functional analysis of leukemia‐associated PTPN11 mutations in primary hematopoietic cells. Blood, 106(1), 311–317. 10.1182/blood-2004-11-4207 15761018 PMC1895116

[ajmgc32012-bib-0096] Schubbert, S. , Zenker, M. , Rowe, S. L. , Böll, S. , Klein, C. , Bollag, G. , … Kratz, C. P. (2006). Germline KRAS mutations cause Noonan syndrome. Nature Genetics, 38(3), 331–336. 10.1038/ng1748 16474405

[ajmgc32012-bib-0097] Simanshu, D. K. , Nissley, D. V. , & McCormick, F. (2017). RAS proteins and their regulators in human disease. Cell, 170(1), 17–33. 10.1016/j.cell.2017.06.009 28666118 PMC5555610

[ajmgc32012-bib-0098] Steklov, M. , Pandolfi, S. , Baietti, M. F. , Batiuk, A. , Carai, P. , Najm, P. , … Sablina, A. A. (2018). Mutations in LZTR1 drive human disease by dysregulating RAS ubiquitination. Science, 362(6419), 1177–1182. 10.1126/science.aap7607 30442762 PMC8058620

[ajmgc32012-bib-0099] Stowe, I. B. , Mercado, E. L. , Stowe, T. R. , Bell, E. L. , Oses‐Prieto, J. A. , Hernandez, H. , … McCormick, F. (2012). A shared molecular mechanism underlies the human rasopathies Legius syndrome and Neurofibromatosis‐1. Genes & Development, 26(13), 1421–1426. 10.1101/gad.190876.112 22751498 PMC3403010

[ajmgc32012-bib-0100] Suzuki, H. , Takenouchi, T. , Uehara, T. , Takasago, S. , Ihara, S. , Yoshihashi, H. , & Kosaki, K. (2019). Severe Noonan syndrome phenotype associated with a germline Q71R MRAS variant: A recurrent substitution in RAS homologs in various cancers. American Journal of Medical Genetics. Part A, 179(8), 1628–1630. 10.1002/ajmg.a.61261 31173466

[ajmgc32012-bib-0101] Tajan, M. , de Rocca Serra, A. , Valet, P. , Edouard, T. , & Yart, A. (2015). SHP2 sails from physiology to pathology. European Journal of Medical Genetics, 58(10), 509–525. 10.1016/j.ejmg.2015.08.005 26341048

[ajmgc32012-bib-0102] Tajan, M. , Paccoud, R. , Branka, S. , Edouard, T. , & Yart, A. (2018). The RASopathy family: Consequences of germline activation of the RAS/MAPK pathway. Endocrine Reviews, 39(5), 676–700. 10.1210/er.2017-00232 29924299

[ajmgc32012-bib-0103] Taniguchi, K. , Kohno, R. , Ayada, T. , Kato, R. , Ichiyama, K. , Morisada, T. , … Yoshimura, A. (2007). Spreds are essential for embryonic lymphangiogenesis by regulating vascular endothelial growth factor receptor 3 signaling. Molecular and Cellular Biology, 27(12), 4541–4550. 10.1128/MCB.01600-06 17438136 PMC1900061

[ajmgc32012-bib-0104] Tartaglia, M. , & Gelb, B. D. (2005). Germ‐line and somatic PTPN11 mutations in human disease. European Journal of Medical Genetics, 48(2), 81–96. 10.1016/j.ejmg.2005.03.001 16053901

[ajmgc32012-bib-0105] Tartaglia, M. , & Gelb, B. D. (2010). Disorders of dysregulated signal traffic through the RAS‐MAPK pathway: Phenotypic spectrum and molecular mechanisms. Annals of the new York Academy of Sciences, 1214, 99–121. 10.1111/j.1749-6632.2010.05790.x 20958325 PMC3010252

[ajmgc32012-bib-0106] Tartaglia, M. , Gelb, B. D. , & Zenker, M. (2011). Noonan syndrome and clinically related disorders. Best Practice & Research. Clinical Endocrinology & Metabolism, 25(1), 161–179. 10.1016/j.beem.2010.09.002 21396583 PMC3058199

[ajmgc32012-bib-0107] Tartaglia, M. , Kalidas, K. , Shaw, A. , Song, X. , Musat, D. L. , van der Burgt, I. , … Gelb, B. D. (2002). PTPN11 mutations in Noonan syndrome: Molecular spectrum, genotype–phenotype correlation, and phenotypic heterogeneity. American Journal of Human Genetics, 70(6), 1555–1563. 10.1086/340847 11992261 PMC379142

[ajmgc32012-bib-0108] Tartaglia, M. , Martinelli, S. , Cazzaniga, G. , Cordeddu, V. , Iavarone, I. , Spinelli, M. , … Biondi, A. (2004). Genetic evidence for lineage‐related and differentiation stage‐related contribution of somatic PTPN11 mutations to leukemogenesis in childhood acute leukemia. Blood, 104(2), 307–313. 10.1182/blood-2003-11-3876 14982869

[ajmgc32012-bib-0109] Tartaglia, M. , Martinelli, S. , Iavarone, I. , Cazzaniga, G. , Spinelli, M. , Giarin, E. , … Biondi, A. (2005). Somatic PTPN11 mutations in childhood acute myeloid leukaemia. British Journal of Haematology, 129(3), 333–339. 10.1111/j.1365-2141.2005.05457.x 15842656

[ajmgc32012-bib-0110] Tartaglia, M. , Martinelli, S. , Stella, L. , Bocchinfuso, G. , Flex, E. , Cordeddu, V. , … Gelb, B. D. (2006). Diversity and functional consequences of germline and somatic PTPN11 mutations in human disease. American Journal of Human Genetics, 78(2), 279–290. 10.1086/499925 16358218 PMC1380235

[ajmgc32012-bib-0111] Tartaglia, M. , Mehler, E. L. , Goldberg, R. , Zampino, G. , Brunner, H. G. , Kremer, H. , … Gelb, B. D. (2001). Mutations in PTPN11, encoding the protein tyrosine phosphatase SHP‐2, cause Noonan syndrome. Nature Genetics, 29(4), 465–468. 10.1038/ng772 11704759

[ajmgc32012-bib-0112] Tartaglia, M. , Niemeyer, C. M. , Fragale, A. , Song, X. , Buechner, J. , Jung, A. , … Gelb, B. D. (2003). Somatic mutations in PTPN11 in juvenile myelomonocytic leukemia, myelodysplastic syndromes and acute myeloid leukemia. Nature Genetics, 34(2), 148–150. 10.1038/ng1156 12717436

[ajmgc32012-bib-0113] Tartaglia, M. , Pennacchio, L. A. , Zhao, C. , Yadav, K. K. , Fodale, V. , Sarkozy, A. , … Gelb, B. D. (2006). Gain‐of‐function SOS1 mutations cause a distinctive form of Noonan syndrome. Nature Genetics, 39(1), 75–79. 10.1038/ng1939 17143282

[ajmgc32012-bib-0114] Tidyman, W. E. , & Rauen, K. A. (2016). Pathogenetics of the RASopathies. Human Molecular Genetics, 25(R2), R123–R132. 10.1093/hmg/ddw191 27412009 PMC6283265

[ajmgc32012-bib-0115] Umeki, I. , Niihori, T. , Abe, T. , Kanno, S. I. , Okamoto, N. , Mizuno, S. , … Aoki, Y. (2019). Delineation of LZTR1 mutation‐positive patients with Noonan syndrome and identification of LZTR1 binding to RAF1‐PPP1CB complexes. Human Genetics, 138(1), 21–35. 10.1007/s00439-018-1951-7 30368668

[ajmgc32012-bib-0116] Ünal, E. B. , Uhlitz, F. , & Blüthgen, N. (2017). A compendium of ERK targets. FEBS Letters, 591(17), 2607–2615. 10.1002/1873-3468.12740 28675784

[ajmgc32012-bib-0117] van der Burgt, I. , & Brunner, H. (2000). Genetic heterogeneity in Noonan syndrome: Evidence for an autosomal recessive form. American Journal of Medical Genetics, 94(1), 46–51. 10.1002/1096-8628(20000904)94:1<46::aid-ajmg10>3.0.co;2-i 10982482

[ajmgc32012-bib-0118] Wakioka, T. , Sasaki, A. , Kato, R. , Shouda, T. , Matsumoto, A. , Miyoshi, K. , … Yoshimura, A. (2001). Spred is a Sprouty‐related suppressor of Ras signalling. Nature, 412(6847), 647–651. 10.1038/35088082 11493923

[ajmgc32012-bib-0119] Wallace, M. R. , Marchuk, D. A. , Andersen, L. B. , Letcher, R. , Odeh, H. M. , Saulino, A. M. , … Collins, F. S. (1990). Type 1 neurofibromatosis gene: Identification of a large transcript disrupted in three NF1 patients. Science, 249(4965), 181–186. 10.1126/science.2134734 2134734

[ajmgc32012-bib-0120] Watkins, F. , Fidler, C. , Boultwood, J. , & Wainscoat, J. S. (2004). Mutations in PTPN11 are rare in adult myelodysplastic syndromes and acute myeloid leukemia. American Journal of Hematology, 76(4), 417. 10.1002/ajh.20134 15282682

[ajmgc32012-bib-0121] Weber, S. M. , & Carroll, S. L. (2021). The role of R‐Ras proteins in Normal and pathologic migration and morphologic change. The American Journal of Pathology, 191(9), 1499–1510. 10.1016/j.ajpath.2021.05.008 34111428 PMC8420862

[ajmgc32012-bib-0122] Weinstock, N. I. , & Sadler, L. (2022). The RRAS2 pathogenic variant p.Q72L produces severe Noonan syndrome with hydrocephalus: A case report. American Journal of Medical Genetics. Part A, 188(1), 364–368. 10.1002/ajmg.a.62523 34648682

[ajmgc32012-bib-0123] Yamamoto, G. L. , Aguena, M. , Gos, M. , Hung, C. , Pilch, J. , Fahiminiya, S. , … Bertola, D. R. (2015). Rare variants in SOS2 and LZTR1 are associated with Noonan syndrome. Journal of Medical Genetics, 52(6), 413–421. 10.1136/jmedgenet-2015-103018 25795793

[ajmgc32012-bib-0124] Yan, W. , Markegard, E. , Dharmaiah, S. , Urisman, A. , Drew, M. , Esposito, D. , … Simanshu, D. K. (2020). Structural insights into the SPRED1‐neurofibromin‐KRAS complex and disruption of SPRED1‐neurofibromin interaction by oncogenic EGFR. Cell Reports, 32(3), 107909. 10.1016/j.celrep.2020.107909 32697994 PMC7437355

[ajmgc32012-bib-0125] Yoon, S. , & Seger, R. (2006). The extracellular signal‐regulated kinase: Multiple substrates regulate diverse cellular functions. Growth Factors, 24(1), 21–44. 10.1080/02699050500284218 16393692

[ajmgc32012-bib-0126] Young, L. C. , Hartig, N. , Boned Del Río, I. , Sari, S. , Ringham‐Terry, B. , Wainwright, J. R. , … Rodriguez‐Viciana, P. (2018). SHOC2‐MRAS‐PP1 complex positively regulates RAF activity and contributes to Noonan syndrome pathogenesis. Proceedings of the National Academy of Sciences of the United States of America, 115(45), E10576–E10585. 30348783 10.1073/pnas.1720352115 PMC6233131

[ajmgc32012-bib-0127] Young, L. C. , & Rodriguez‐Viciana, P. (2018). MRAS: A close but understudied member of the RAS family. Cold Spring Harbor Perspectives in Medicine, 8(12), a033621. 10.1101/cshperspect.a033621 29311130 PMC6280710

[ajmgc32012-bib-0128] Zenker, M. , Buheitel, G. , Rauch, R. , Koenig, R. , Bosse, K. , Kress, W. , … Rauch, A. (2004). Genotype‐phenotype correlations in Noonan syndrome. The Journal of Pediatrics, 144(3), 368–374. 10.1016/j.jpeds.2003.11.032 15001945

[ajmgc32012-bib-0129] Zhao, X. , Li, Z. , Wang, L. , Lan, Z. , Lin, F. , Zhang, W. , & Su, Z. (2021). A Chinese family with Noonan syndrome caused by a heterozygous variant in LZTR1: A case report and literature review. BMC Endocrine Disorders, 21(1), 2. 10.1186/s12902-020-00666-6 33407364 PMC7788825

